# Arthroscopically assisted fixation of the lesser trochanter fracture: a case series

**DOI:** 10.1093/jhps/hnu006

**Published:** 2014-08-22

**Authors:** Aditya Khemka, Guy Raz, Belinda Bosley, Gerdesmeyer Ludger, Munjed Al Muderis

**Affiliations:** 1. Norwest Private Hospital, Bella Vista 2153, Sydney, Australia 2. Department of Joint Arthroplasty and Clinical Science, Mare Clinic, Universitatsklinikum Schleswig-Holstein, Kiel, Germany 3. University Of Notre Dame, Australia School of Medicine, Sydney, Australia

## Abstract

Avulsion fractures of the lesser trochanter in adolescents are uncommon. This injury is a result of a sudden forceful contraction of the iliopsoas tendon. It usually occurs during vigorous sport activity. Historically, these injuries were treated non-operatively, with guarded results, including weak hip flexor strength and non-union, hindering return to competitive sport. We report a series of three arthroscopically assisted fracture fixations performed by the senior author, using cannulated screw fixation in two cases and an anchor in one case. Mobilization was commenced immediately following surgery, allowing weight bearing as tolerated using crutches for 4 weeks, thereafter unaided walking was allowed. Patients were assessed at 2 weeks, 6 weeks, 3 months and 1-year post-operatively. Radiographs were utilized to confirm full union. All three patients were able to mobilize unaided by 4 weeks post-operatively and two of the three patients returned to competitive sport at 3 months. Near—anatomical union was achieved in all cases. No complications were noted during surgery and the peri-operative period in our series. The utilization of arthroscopic reduction and fixation of avulsion of the lesser trochanter results in good fixation and allows a faster recovery with a return to sports activity, and therefore, we suggest it as a viable treatment option for such injuries.

## INTRODUCTION

Injuries to the hip and groin related to athletic activities account for 1 in 10 patient visits to sports clinics [1–3]. Sudden and forceful muscle contraction can result in avulsion fractures of the apophysis in adolescents while similar mechanisms of injury may lead to muscle sprains in adults [4, 5].

The apophysis is an osteochondral plate at the tendinous insertion. It is a secondary centre of ossification contributing to a change in shape or size of the bone, but not its length [6]. Milch [7] suggested that injuries to the apophysis were similar in nature to epiphyseal injuries and he called them apophysiolysis. In adolescents the muscles are more resistant to injury than the bones [5, 8]. This imbalance renders the apophysis to be the weakest part of the skeleton, and makes it vulnerable to avulsion injuries [4, 5, 8].

Salter and Harris described two types of epiphysis in their original article [9]. A traction epiphysis is the site of the insertion or origin of a major muscle or muscle group, and its weakest point is the epiphyseal plate because the Sharpey’s fibres attaching the muscle to the epiphysis are stronger than the junction of cells between the calcified and uncalcified epiphysis [9]. This is the basis of avulsion injuries of the apophysis.

**Figure 1.**
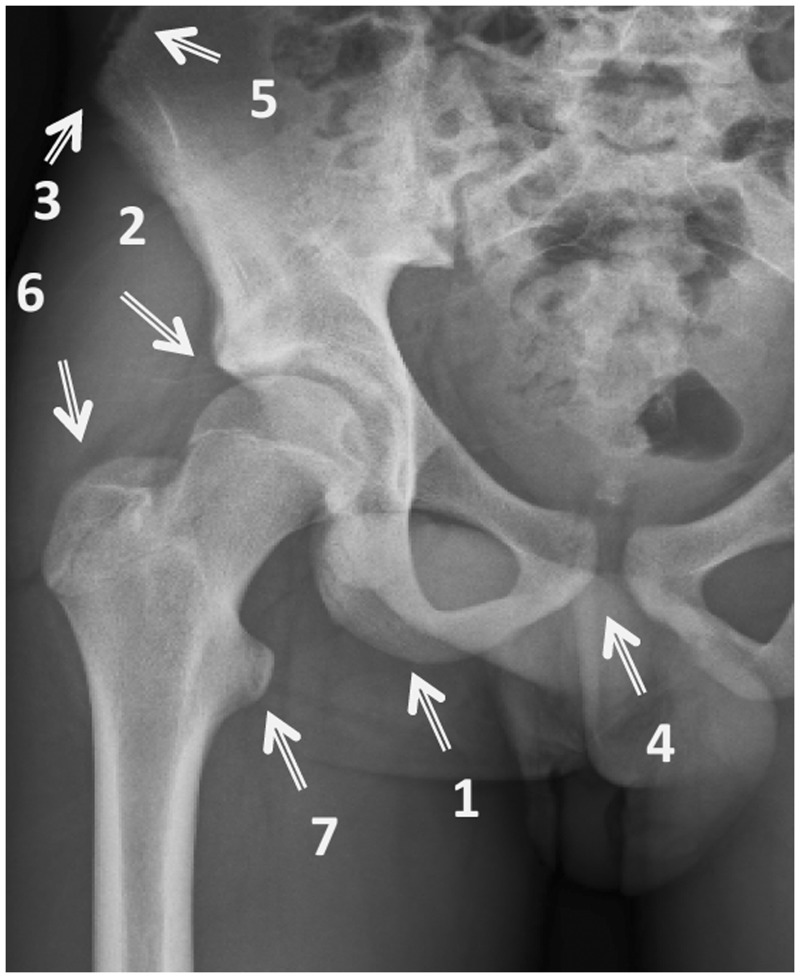
Sites for avulsion injuries in the pelvis and proximal femur. [10].

Avulsion injuries at the pelvis and proximal femur have been previously described in the literature [5, 10]. Sites that can sustain avulsion fractures have been identified and are demonstrated in Fig. 1 [5, 10].

Ischial tuberosity (avulsion of hamstrings), the anterior inferior iliac spine (avulsion of the rectus femoris), and the anterior superior iliac spine (avulsion of the sartorius) are the common injuries [4, 8, 10, 11]. Avulsion fractures of the lesser trochanter, greater trochanter and iliac crest are rare injuries [4, 5, 8, 10, 11]. Isolated avulsion fractures of the lesser trochanter represent <1% of hip injuries in the orthopaedic surgeon’s practice [4].

Historically, isolated avulsion fractures of the lesser trochanter have been managed conservatively with guarded results [4, 8, 11–14]. For the first time in the literature, we report arthroscopically assisted operative fixation of three cases of isolated avulsion fractures of the lesser trochanter when it is still in the apophyseal stage in adolescence between August 2010 and August 2013. Each of these cases was referred by their primary treating orthopaedic surgeon for consideration of surgical treatment.

## CASE REVIEW (METHODS)

Three patients presented to our clinic between August 2010 and August 2013 with an isolated avulsion fracture of the lesser trochanter. All three cases resulted from vigorous physical activity, in male adolescents. The exact mechanism of injury was difficult to determine, but in all instances, sprinting was involved (Table I).

**Table I.** Demographic findings

	Case 1	Case 2	Case 3
Age (yrs)	15	16	15
Sport	Rugby	Soccer	Soccer
Timing	Chronic	Acute	Acute

Clinical presentation was that of a teenage male athlete, experiencing a sudden, sharp pain in the groin while sprinting. The pain in most cases was severe enough to discontinue the activity immediately, except on one occasion when he could continue running for a few minutes before the soreness was unbearable. In all instances, patients lost the ability to flex the hip and to walk upstairs in normal sequence.

On examination, all the patients walked with an antalgic gait. There was no distress at rest, but they avoided movement of the involved leg. There was severe tenderness on direct palpation without any obvious swelling. In the supine position, no patient could perform a straight leg raise. Rolling of the leg while in full extension did not elicit any tenderness. In the sitting position (with hips flexed at 90°), pain was elicited with further flexion of the hip. All patients had the tendency to position the hip in a semi-flexed externally rotated position compared with the normal side.

**Figure 2.**
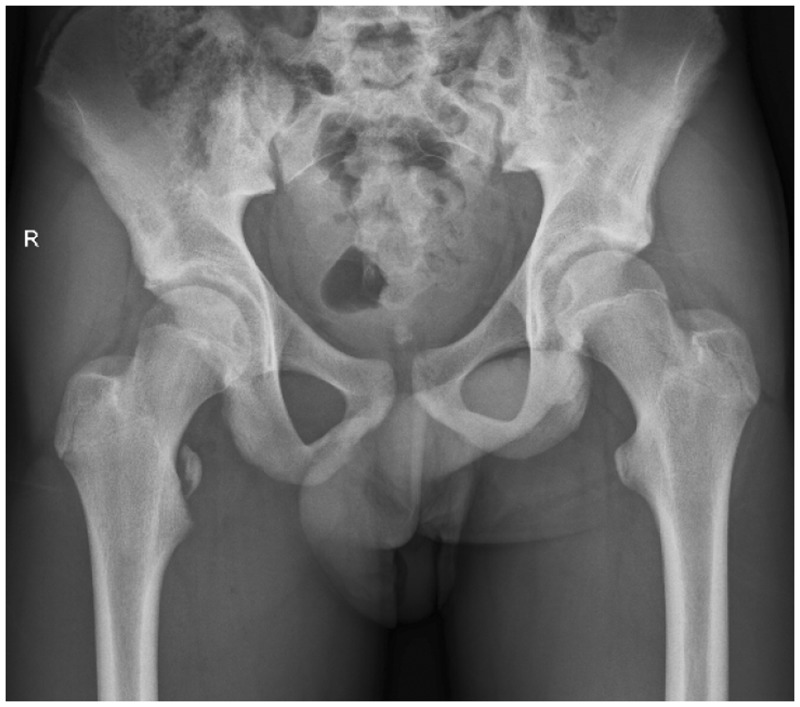
Plain radiograph showing an avulsion of the right lesser trochanter.

**Figure 3.**
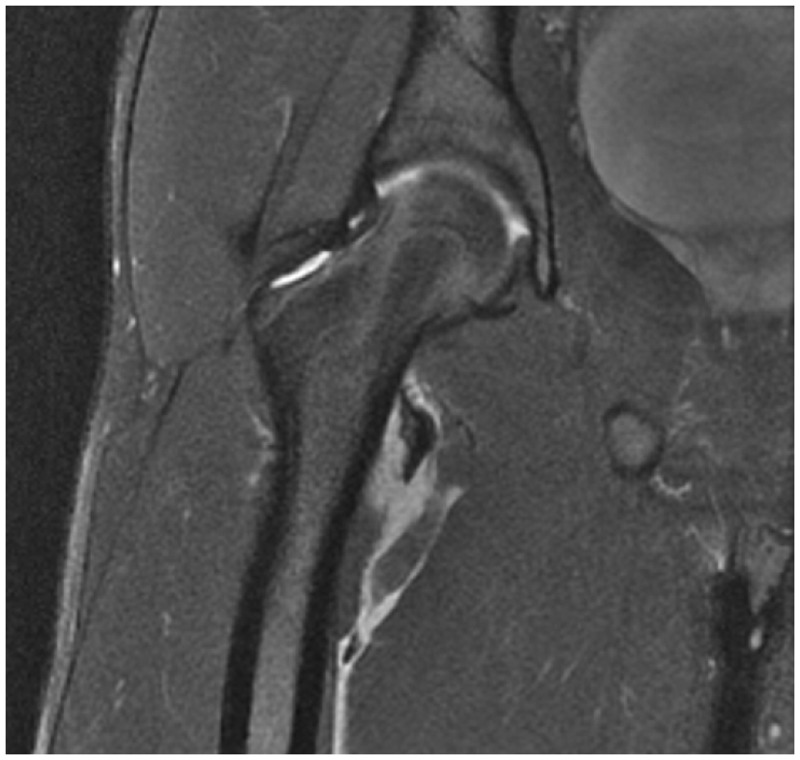
MRI scan showing an avulsion of the right lesser trochanter.

A plain anteroposterior (AP) radiograph of the pelvis demonstrated an avulsed fragment (Fig. 2). Plain radiographs also aided us to delineate the degree of the displacement of the avulsed fragment. Magnetic resonance imaging (MRI) scan was used in all three cases to assess the condition of the soft tissue associated with the avulsion of the fragments (Fig. 3).

On investigation, we found that all of our patients had isolated avulsion fractures of the lesser trochanter with a displacement of >2 cm. There was a significant amount of soft tissue oedema around the lesser trochanter in association with capsular injury and a partial tear of the iliopsoas tendon at the lesser trochanter. There was no evidence of heterotrophic ossification related to the displaced fragment.

**Figure 4.**
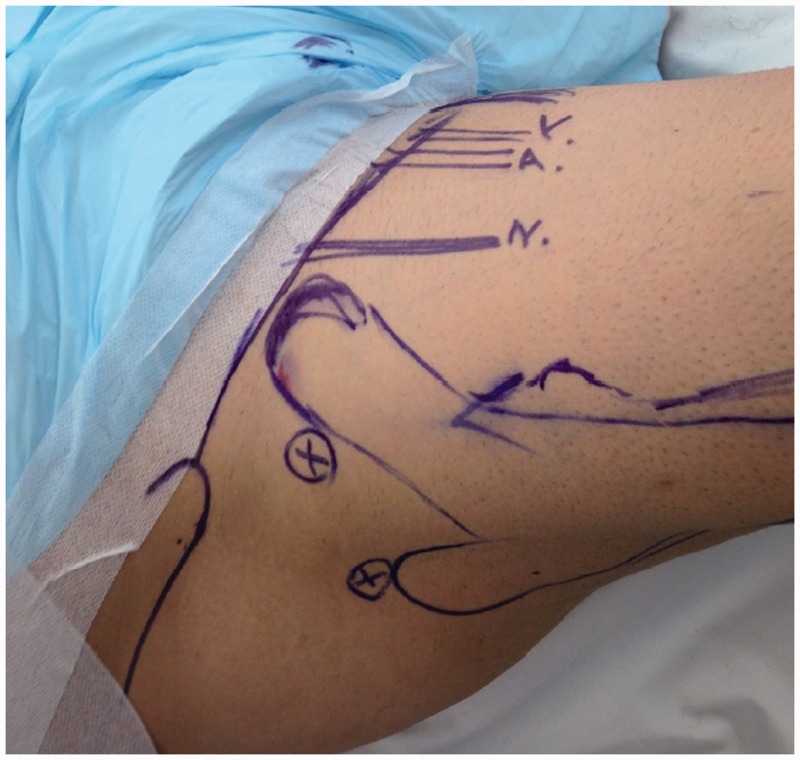
Lateral and anterolateral portals used for hip arthroscopy.

**Figure 5.**
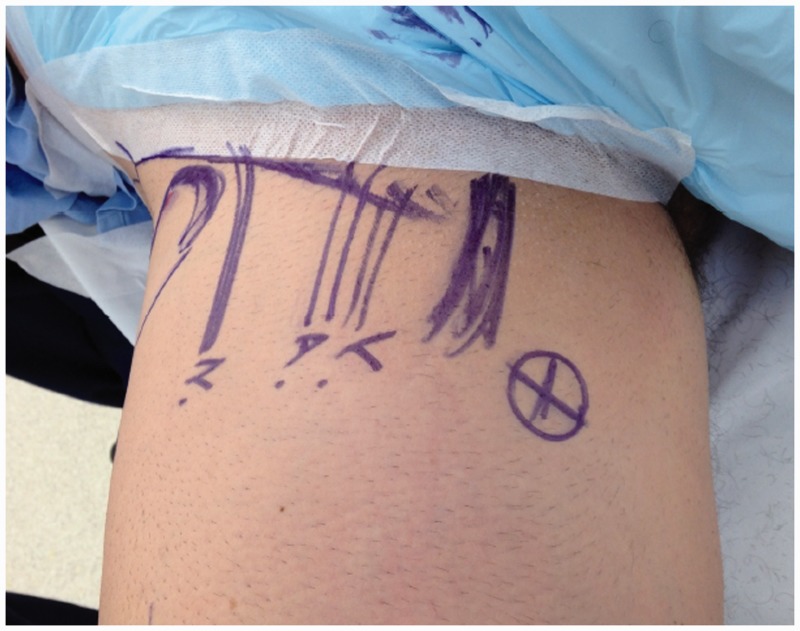
Medial portals used for hip arthroscopy.

**Figure 6.**
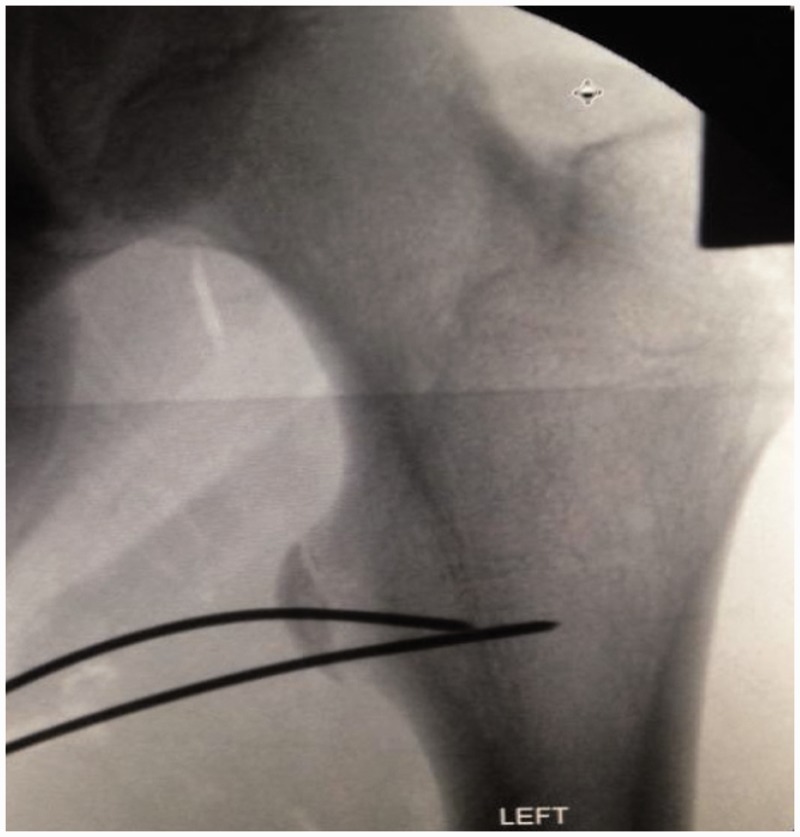
K-wire used to hold fragment *in situ*.

**Figure 7.**
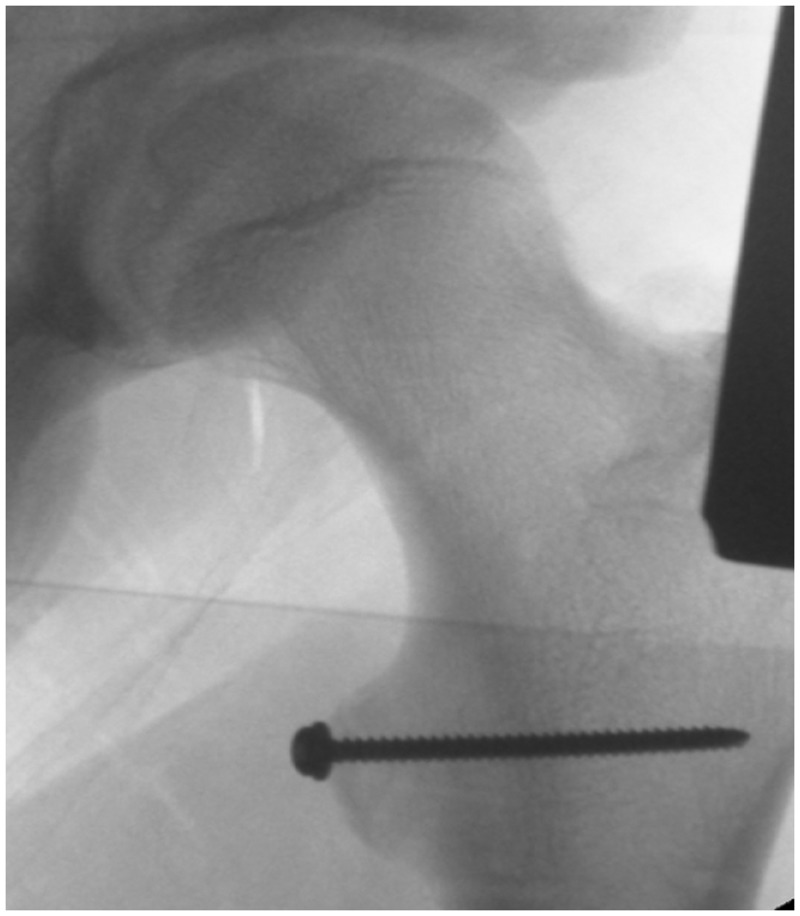
Cancellous screw fixation.

**Figure 8.**
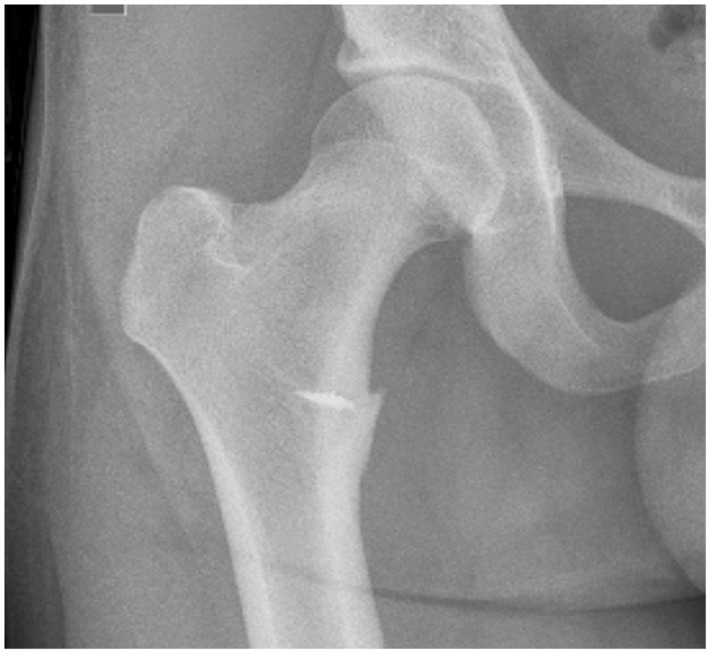
Fixation using a suture anchor.

All patients underwent arthroscopically assisted internal fixation to re-attach the avulsed fragment and the tendon of the iliopsoas onto the lesser trochanter. This was performed under general anaesthesia. For the arthroscopy we used a standard radiolucent table without traction. The patients were positioned supine. Standard preparation and draping using hip replacement protocols for a Ludloff’s approach were used [15, 16]. A standard lateral portal was used for the camera, an anterolateral portal for instrumentation and an additional medial portal was used to place an arthroscopic cannula through which the fixation was achieved [17–20]. The lateral portal was located 2 cm anterior and 1–2 cm superior to the tip of the greater trochanter (Fig. 4). The anterolateral portal was placed at the intersection of perpendicular lines drawn laterally from the superior aspect of the symphysis pubis and inferiorly from the anterior superior iliac spine (Fig. 4). The medial portal was placed 5–6 cm below the pubic tubercle just medial to adductor longus (Fig. 5). Intra-operatively the hip joint was not distracted. Instruments were inserted intra-capsularly but extra-articularly, anteriorly in relation to the femoral neck. The hip joint manifested a large effusion in the acute cases, which was lavaged. The fluid pump was maintained at a minimum flow and at a pressure of 60 mmHg to minimize extravasation of fluid into the surrounding tissues. Visualization of the fracture was achieved after minimal dissection of the capsule medially using an arthroscopic shaver and tissue ablation wand through the tear in the capsule at its attachment just proximal to the lesser trochanter. The fracture fragment was then held using a pituitary rongeur (5 mm), the patient’s leg was then positioned in maximum external rotation and at 45° flexion. The fracture fragment was then manoeuvred into near anatomical position and held *in* *situ* with K-wires. The position of the reduction was then checked with an image intensifier (Fig. 6). A cannulated screw was passed over the most vertical wire holding the fracture in place in two of the cases while a 5.5-mm anchor was used to secure the chronic fragment due to its small size. There was no evidence of any heterotrophic ossification around the fragment. The insertion of the wires, screws and anchor was done through the medial portal, which lay directly against the lesser trochanter (Figs. 7 and 8). The wound was closed using single interrupted sutures (3-0 Monocryl) after local anaesthetic infiltration.

## RESULTS

There were no peri-operative complications. All three cases were hospitalized for 48 h. They were treated with parenteral antibiotics for a day. Pain was well controlled with oral analgesics. We did not use any form of casting or bracing. Crutch weight bearing commenced immediately, the day following the surgery. Plain radiographs were taken on the day after surgery and showed excellent alignment. Weight bearing as tolerated with crutches was advised for a period of 4 weeks with precautions to avoid active flexion and no straight leg raise. Physiotherapy to strengthen their hip flexors was started following this period. All the patients were allowed to resume sporting activity 10–12 weeks after surgery. All the patients were reviewed 2, 6 and 10 weeks later. At every follow-up, they were assessed for pain, range of motion in the hip and hip radiographs. All the cases had an excellent range of motion at the hip and the radiographs showed excellent alignment and healing. The mean follow-up was 16 months (range, 12–36 months). The two patients with acute injuries successfully returned to impact activity, which involved sprinting.

We had one case of numbness on the medial side of the knee (anterior femoral cutaneous nerve of the thigh). The numbness was transient and subsided within two weeks of surgery.

## DISCUSSION

A lesser trochanter avulsion fracture is a rare injury representing <1% of hip injuries [4, 14]. Similar to other avulsion fractures, it is the result of a sudden forceful muscle contraction. In young athletes, it is usually a result of a forceful contraction whilst in adults this injury is typically indicative of an underlying malignancy [21]. Speed notes that this injury usually occurs in boys whilst playing running games [22].

Our literature review suggests that even when large and displaced avulsion fractures are managed non-operatively there are guarded results [4, 5, 14]. Rest, non-steroidal anti-inflammatory medications and non-weight bearing have been historically recommended [4, 5, 8, 14]. Treatment with immobilization in a spica cast has been used but has been found unnecessary given the satisfactory results obtained with symptomatic management [11]. Metzmaker and Pappas treated 27 avulsion fractures using a five-phase protocol with return to sport activity after 12–16 weeks [11].

Surgical repair with open reduction and internal fixation has been reported in cases where conservative management has resulted in non-union [23, 24].

Our review revealed no case reports documenting operative treatment of acute apophyseal avulsion fracture of the lesser trochanter. However, surgical intervention has been indicated in certain instances as reported by Sundar and Carty [25]. They found a limitation of sporting ability in 50% of the cases reviewed with persistent symptoms in 30% of the patients, mostly in those with ischial avulsion injuries [25]. Many authors in the past have described displacement of >2 cm, painful non-union, exostosis and inability to return to sport as indications for surgery [26–31]. Martin and Pipkin in 1957 classified ischial tuberosity avulsion fractures into three groups: non-displaced fractures, acute avulsion fractures and non-union but they did not account for displacement [32]. McKinney and Nelson used displacement as a tool to classify the avulsion fractures and they felt that this helped them in determining the need for surgery [33] (Table II).

**Table II.** Classification of avulsion fractures [33]

Type 1	Undisplaced
Type 2	<2 cm
Type 3	>2 cm
Type 4	Non-union

In our opinion, displaced avulsion fragments bring two major concerns to the fore when considering non-operative treatment. The first is non-union and the second is loss of strength secondary to muscle shortening [33]. There have been arguments in the literature that shortening in muscle length has eventually regained muscle strength equivalent to the contralateral unoperated side. However, none has been advanced for avulsion injuries of the lesser trochanter [11].

Ischiofemoral impingement (IFI) was first described by Johnson in 1977 [34–36]. He suggested the distance between the lesser trochanter and the ischium might be reduced as a result of previous fracture or surgery. This was treated with injection of local anaesthetic around the lesser trochanter, and followed by excision of the lesser trochanter with a satisfactory outcome in all his patients [34]. Wasting or oedema of the quadratus femoris muscle has commonly been attributed to the condition [35] MRI demonstrates inflammation and oedema in this space and quadratus femoris, and can be differentiated from an acute tear [37]. Our concern is that a displaced lesser trochanter >20 mm may potentially lead to an IFI, especially in the presence of heterotrophic ossification [34]. Stafford and Villar [34] through their article suggested peri-articular decompression using arthroscopy to access the ischiofemoral space.

In our opinion, an arthroscopically assisted fixation of such injuries minimizes the chances of a non-union as well as loss of muscle strength and potentially IFI. The choice of internal fixation would be dependent on the shape and location of the fragment. The minimally invasive approach reduces the risk of damage to the neurovascular structures (anterior division of the obturator nerve and medially the femoral vessels and nerves) and avoids the other complications of open surgery but requires a high level of skill and experience to conduct.

## CONCLUSION

The approach to the evaluation of musculoskeletal injuries in adolescents requires consideration of the peculiarities of the immature skeleton. In principle, hip arthroscopy provides a minimally invasive technique that can explore wider areas of the hip joint with minimal risk of tissue damage. Indications to use hip arthroscopy are rapidly expanding. In the hands of a trained hip arthroscopy surgeon, internal fixation of a lesser trochanter fracture can be safely achieved. We suggest this technique is safe, reproducible and reliable.

## FUNDING

None of the authors have received any form of financial support in contribution to produce this article.

## CONFLICT OF INTEREST STATEMENT

None declared.
